# Strengthening resilience to address climate change and chronic diseases: evidence from small states

**DOI:** 10.1093/eurpub/ckac129.222

**Published:** 2022-10-25

**Authors:** S Moncada, M Spiteri

**Affiliations:** Islands and Small States Institute, University of Malta, Msida, Malta

## Abstract

**Background:**

Climate & environmental change are disproportionally impacting small states, given higher costs per capita associated with coping with the immediate risks of environmental & climatic events. Climate change has been identified as a serious public health threat. This is especially true for heatwaves in the Mediterranean Basin, with poor air quality worsening the health impacts during periods of extreme heat, often affecting already high levels of chronic diseases. Promoting climate change adaptation measures is crucial to address the negative socio-economic impacts brought by heatwaves, & its interactions with poor air quality. While awareness about the link between poor air quality & heat-waves is gaining momentum, there is still a gap in policy responses, especially in small states. This research assesses the level of preparedness of the European Union, with a focus on adaptation to heat-waves since the extreme European heat-wave of 2003. A case study on Malta is also presented, aiming at discovering what measures the island is adopting to tackle the problems arising from the interaction between poor air quality & heatwaves.

**Methods:**

A systematic literature review is conducted, investigating the links between heat-waves & air pollution post 2003, followed by a qualitative content analysis to assess the preparedness of climate change adaptation measures in this regard.

**Results and conclusions:**

The findings of this research show that the link between heat waves & poor air quality has not been sufficiently acknowledged by academia, with evident gaps in applied small states research. Also, a closer look at key adaptation policies & measures in Malta finds that heat waves & poor air quality are hardly been linked, leaving much scope to introduce policy & economic instruments to tackle both public health risks to address growing chronic diseases, & not to compromise current & future socioeconomic wellbeing.

